# Symmetry-based derivation of neuronal membrane dynamics: a Lie group approach to action potential generation

**DOI:** 10.3389/fnins.2026.1896585

**Published:** 2026-09-03

**Authors:** Robert F. Melendy, Daniel H. Blue

**Affiliations:** George Fox University, Department of Electrical Engineering and Computer Science, Newberg, OR, United States

**Keywords:** action potential, Casimir invariants, circuit synthesis, Lie groups, membrane dynamics, neuromorphic circuits, symmetry principles

## Abstract

We derive the nonlinear dynamics of neuronal membrane potential from fundamental physical symmetries using Lie group theory. Starting from three experimentally verified symmetries [rotational invariance of paramagnetic ion (Ca^2 +^, Na^+^) orientation producing SO(2), scale invariance of membrane conductance ratios producing ℝ, and time-translation symmetry producing ℝ], we prove that these independent physical observations uniquely determine the semidirect product group structure *G* = SO(2) ⋉ℝ^2^, as established in Theorem 1.4. The structure constants (γ_1_ = 1.58 V/s, γ_2_ = 0.025 V^−1^, γ_3_ = 0.003) derive from independent measurements in cable theory, Hodgkin–Huxley voltage gating, and Faraday induction, establishing the complete Lie algebra without reference to any phenomenological action potential model. Despite the presence of these physical symmetries, existing neuromorphic models lack a rigorous mathematical framework that systematically predicts functional forms from first principles, relying instead on empirical fitting or stochastic approximations that obscure the underlying deterministic structure. From the group structure *G* = SO(2) ⋉ℝ^2^, we derive three mathematical constraints on membrane voltage: SO(2) compactness requires bounded functions (tanh, 1/cosh), scale invariance mandates power-law terms (*x*^ξ^), and temporal symmetry requires oscillatory behavior [sin(π*x*/τ_0_)]. These constraints predict a unique functional form V(x) ∼ *x*^ξ^ ⋅sin(π*x*/τ_0_)/[*G*(x)⋅cosh(π*x*/τ_0_)⋅*f*(*x*)]. Remarkably, our previously published phenomenological equation (Melendy, 2018) (discovered through empirical fitting before developing the present theoretical framework) exhibits exactly this predicted structure, validating that the Lie group approach captures the fundamental physics governing neuronal excitability. This represents theory predicting phenomenology, not *post-hoc* rationalization. The symmetries were identified from independent physical principles, the group structure was mathematically derived, and the functional form was predicted: all without reference to the 2018 empirical equation. The subsequent agreement demonstrates that the 2018 equation was not arbitrary phenomenological fitting but rather discovered the unique form required by underlying symmetry principles. We present a deterministic algorithm for neuromorphic circuit synthesis wherein Lie algebra structure maps to circuit topology, with components as physical realizations of group exponentials and Kirchhoff’s laws emerging as Casimir constraints. The framework determines concrete physical predictions: the power-law exponent ξ≈ e/2 ≈ 1.359, obtained from Lyapunov trajectory separation analysis, characterizes depolarization growth rate and emerges naturally from the algebraic structure. Comparison with classical Hodgkin–Huxley dynamics demonstrates that the simplified Lie group equation captures physiologically realistic action potential waveforms while revealing why the functional form takes its specific mathematical structure. All transcendental functions employ proper dimensional scaling through characteristic time τ_0_, ensuring mathematical rigor. This framework offers a principled foundation for neuromorphic circuit design, bridging mathematical physics and engineering to enable reproducible, scalable, and symmetry-preserving models of neuronal excitability. By grounding neuromorphic systems in fundamental symmetry principles rather than phenomenological approximations, this approach enables systematic circuit synthesis with physical consistency guaranteed by the algebraic structure.

## 1 Introduction

### 1.1 The problem: phenomenology vs. principle

The Hodgkin–Huxley (HH) formalism (Hodgkin and Huxley, 1952; Hodgkin and Katz, 1949) successfully describe action potential generation through four coupled nonlinear differential equations governing ion channel kinetics. However, these equations are fundamentally phenomenological (i.e., they are fitted to experimental data without deeper theoretical justification for their mathematical form).

In previous work (Melendy, 2015, 2016, 2018), we derived a single closed-form equation for membrane potential *V*_*m*_ by synthesizing three biophysical factors: cable conductance (Jack et al., 1975; Pethig and Kell, 1987; Rall, 1977), paramagnetic response (Kittel, 2004; Varfolomeev et al., 1976), and field modulation (Greenebaum and Barnes, 2007; Roth and Wikswo, 1985a,b; Wijesinghe, 2014). While this model achieved 2.3% accuracy, it remained phenomenological – we demonstrated these factors are sufficient but not why they are necessary or how they interact.

We note explicitly that the present framework departs from conventional cable theory and Hodgkin–Huxley formalism in two intentional respects. First, whereas standard cable theory treats axial resistivity as spatially uniform and voltage-independent, the present framework treats membrane conductance ratios as scale-invariant under uniform scaling. This is a deliberate symmetry-based abstraction motivated by the Lie group derivation, not a misapplication of cable theory. Second, the magnetic field effects incorporated here are not claimed as dominant biophysical mechanisms in the conventional electrophysiological sense, but rather as symmetry generators whose mathematical properties constrain the functional form of membrane voltage dynamics. Readers are encouraged to interpret results within the neuromorphic engineering context for which this framework is designed, rather than as a replacement for conventional biophysical modeling.

### 1.2 The symmetry principle approach

Modern physics derives dynamical equations from symmetry principles rather than empirical fitting (Gilmore, 2008; Olver, 1993). Noether’s theorem (Noether, 1918) establishes that every continuous symmetry implies a conserved quantity, and the generators of these symmetries form a Lie algebra whose structure determines the dynamics (Arnold, 1989; Faddeev and Takhtajan, 1987). This approach has revolutionized particle physics, general relativity, and integrable systems (Nakahara, 2003; Weinberg, 1995).

This paper’s central question: *Can we derive neuronal membrane dynamics from physical symmetries and synthesize deterministic neuromorphic circuits from the resulting Lie group structure?*

### 1.3 Background: group theory and neuromorphic circuits

Group theory provides a natural language for describing nonlinear dynamics through symmetries (Gilmore, 2008; Olver, 1993). When a dynamical system possesses continuous symmetries, these symmetries form a Lie group 𝔤, and the infinitesimal generators form a Lie algebra 𝔤. The dynamics can then be understood as flows on the group manifold, with conserved quantities arising from Noether’s theorem (Noether, 1918). This framework has proven powerful in classical mechanics (Arnold, 1989), integrable systems (Faddeev and Takhtajan, 1987), and theoretical physics (Nakahara, 2003; Weinberg, 1995).

Recent applications to neuroscience have explored symmetry in neural field equations and equivariant neural networks, but a systematic Lie group treatment of single-neuron biophysics and its circuit realization has not been developed.

Furthermore, recent computational approaches to neuronal electrical signaling have employed finite volume and finite difference methods to solve phenomenological models with high numerical precision (Saleem et al., 2024). These numerical methods constitute an important and complementary class of methodology for computational neuroscience. *The present manuscript addresses a categorically different problem:* rather than solving known differential equations more accurately, we derive the functional form of those equations from first principles using Lie group theory, and prove that three physical symmetries uniquely constrain both the mathematical form of the voltage equation and the topology of the corresponding neuromorphic circuit. These contributions are complementary rather than competing.

Neuromorphic engineering seeks to emulate neural computation in analog hardware. Traditional approaches either: (1) directly implement HH equations using operational amplifiers and nonlinear elements, requiring extensive calibration; (2) employ phenomenological spiking models (Gerstner and Kistler, 2002; Izhikevich, 2003) that sacrifice biophysical detail for simplicity. Neither approach provides a principled algebraic framework for circuit topology.

The present work is a mathematical proof of concept. It proves that a neuromorphic circuit can be synthesized deterministically from symmetry principles, and that the resulting circuit topology and functional form are uniquely constrained by the Lie algebra structure. The framework reveals symmetry-constrained relations and topological structure rather than independently determining all circuit parameters in isolation. The physical construction and experimental testing of such a circuit constitutes the natural engineering follow-on to this theoretical foundation, as identified explicitly in Future Direction 5.

### 1.4 Contributions of this work

We establish the following:

**Theorem 1.1** (Structural Correspondence). *The nonlinear differential equation for V_*m*_ derived in* Melendy (2018) *is the projection of a flow on a Lie group G = K ⋉ H, where K is a compact subgroup (magnetization) and H is a non-compact subgroup (conductance and modulation).*
**Theorem 1.2** (Circuit-Algebra Equivalence). *Each circuit element corresponds to an exponential map* exp(*α_*i*_X_*i*_*) *of a generator X_*i*_* ∈𝔤, *and Kirchhoff’s laws are equivalent to Casimir constraints.*
**Theorem 1.3** (Stability via Representation Theory). *Bifurcations correspond to eigenvalue crossings in the spectrum of the adjoint representation ad :* 𝔤→ *End*(𝔤).

It’s important to distinguish two categories of claims in this manuscript. The first category comprises experimentally established physical observations: paramagnetic ions possess magnetic moments governed by Langevin orientational statistics (Coffey et al., 1996; Kittel, 2004) and independently measured by Roth and Wikswo (Roth and Wikswo, 1985a,b); membrane conductance ratios remain invariant under uniform scaling, directly observable in Hodgkin–Huxley gating kinetics (Hodgkin and Huxley, 1952); and the action potential exhibits temporal translation invariance, a well-documented electrophysiological property. These are not theoretical assumptions but independently verified physical observations drawn from the existing literature. The second category comprises the theoretical framework derived from these observations: that the three symmetries uniquely determine the semidirect product structure *G* = SO(2) ⋉ℝ^2^, and that this group structure prescribes a deterministic neuromorphic circuit topology. Readers are encouraged to maintain this distinction throughout.

### 1.5 Overview of results

We identify three fundamental symmetries in membrane electrophysiology (rotational, scale, temporal) and derive their Lie algebra structure from Poisson brackets. The group *G* = SO(2) ⋉ℝ^2^ is uniquely determined (Theorem 1.4). We then present Algorithm 1, a deterministic procedure that:

(1) Takes as input: Lie algebra 𝔤 with structure constants $\left\{c_{i\,j}^{k}\right\}$.
(2) Outputs: Circuit netlist with component values and topology.
(3) Guarantees: Kirchhoff’s laws = Casimir constraints; dynamics = group flows.

This is not a metaphor: it is rigorous algebraic circuit synthesis with reproducible implementation.

## 2 Physical symmetries and their generators

### 2.1 Rotational invariance (magnetization)

#### 2.1.1 Physical basis

Paramagnetic ions (primarily Ca^2+^ and Na^+^) in the intracellular space possess magnetic moments μ that interact with external magnetic field *B*. The interaction energy is:


U=-μ⋅B=-μ⁢B⁢cos⁡θ (1)

where θ is the angle between μ and *B*. In thermal equilibrium at temperature *T*, the orientation distribution follows Boltzmann statistics. The mean magnetization is given by the Langevin function (Coffey et al., 1996; Kittel, 2004):


$$M=M_{s\,a\,t}\,ℒ\,\left(\frac{\mu \,B}{k\,T}\right)$$ (2)

where


$$ℒ\,\left(x\right)=\coth\left(x\right)-\frac{1}{x}$$ (3)

and *M*_*sat*_ is the saturation magnetization, *k* is Boltzmann’s constant.

A clarification is warranted regarding the use of the term ‘paramagnetic’ in this context. Na^+^ and Ca^2+^ are diamagnetic in the sense of their ground-state electronic configurations, a fact of atomic physics we do not dispute. The Langevin function employed here describes orientational paramagnetism in the statistical mechanics sense: the thermal alignment of ionic magnetic dipole moments with an external applied field, governed by Boltzmann statistics (Coffey et al., 1996; Kittel, 2004). This treatment applies to nuclear and ionic magnetic moments subject to thermal randomization, regardless of electronic configuration, and is the standard formalism for this class of magnetic response as presented in Kittel (Kittel, 2004). The measured intracellular magnetic fields of Roth and Wikswo (Roth and Wikswo, 1985a,b) provide independent experimental grounding for the field magnitudes used in the derivation of structure constant γ_1_. The reader is encouraged to interpret the paramagnetic response described here within this well-established statistical mechanics framework, not as a claim about electronic paramagnetism.

The increased ionic current density during depolarization produces a corresponding increase in the intracellular magnetic field (Roth and Wikswo, 1985a,b), which in turn drives the magnetization *M* toward saturation. This saturation behavior, governed by the Langevin function, provides the fundamental SO(2) rotational symmetry underlying the bounded amplitude response.

This paramagnetic mechanism, when incorporated into neuronal cable theory with measured membrane magnetic fields (Roth and Wikswo, 1985a,b), successfully reproduces classical Hodgkin–Huxley action potential dynamics with high accuracy (Melendy, 2018), validating the physical relevance of the approach.

#### 2.1.2 Generator derivation

The infinitesimal generator of rotations is:


$$X_{M}=\frac{\partial }{\partial \theta }$$ (4)

For a state vector | ψ⟩ representing ion orientation, the finite rotation by angle *α_*m*_* is:


$$\left|\psi \,\left(\alpha_{M}\right)\right\rangle =\exp\left(\alpha_{m}\,X_{m}\right)\,\left|\psi \,\left(0\right)\right\rangle$$ (5)

Since rotations form a circle (*α_*m*_* ∈ [0, 2π)), the group is *compact*: *K* = SO(2).

#### 2.1.3 Boundedness of magnetization

**Crucial property:** Because SO(2) is compact, magnetization is necessarily bounded, such that:


$$M\in \left[-M_{s\,a\,t},M_{s\,a\,t}\right]$$ (6)

This is not an empirical observation but a mathematical consequence of rotational symmetry. The Langevin function’s saturation ℒ(∞) = 1follows from group compactness.

### 2.2 Scale invariance (conductance)

#### 2.2.1 Physical basis

Consider a membrane with conductances {*G*_1_, *G*_2_, . . ., *G*_*n*_}. If we scale all conductances by factor λ > 0. The conductance generator is which acts on conductance via scaling, such that:


$$G_{i}\to \lambda \,G_{i}\;\forall i$$ (7)

the ratios *G*_*i*_/*G*_*j*_ are preserved. This is scale invariance (’t Hooft, 1973).

#### 2.2.2 Generator derivation

Define the action:


$$G\,\left(\alpha_{c}\right)=e^{\alpha_{c}}\,G_{0}$$ (8)

The infinitesimal generator is:


$$X_{c}=G\,\frac{\partial }{\partial G}$$ (9)

Since *α_*c*_* ∈ (-∞, ∞) and *G* ∈ (0, ∞), the group is *non-compact*, such that *H*_*c*_≅ℝ.

#### 2.2.3 Unboundedness of conductance

**Crucial property:** Because *X*_*c*_ generates the non-compact group ℝ, conductance has no upper bound:


G∈(0,∞) (10)

This unboundedness allows bifurcations where *G* →∞ at threshold.

function’s saturation ℒ(∞) = 1follows from group compactness.

### 2.3 Time modulation

#### 2.3.1 Physical basis

External electric fields **E***_*ext*_*(*t*) modulate ion channels. For periodic stimulation:


$${\text{E}}_{e\,x\,t}\,\left(t\right)\propto \sin\left(\frac{\pi \,t}{\tau_{0}}\right)$$ (11)

where τ_0_ = 1 ms is the characteristic timescale of the action potential (i.e., it is a time normalization constant set by the depolarization-repolarization cycle duration).

#### 2.3.2 Generator derivation

The time-evolution operator is:


$$U\,\left(t\right)=\exp\left[\alpha_{o}\,\left(t\right)\,X_{0}\right]$$ (12)

where the time-modulation generator *X*_*o*_ produces time-dependent parameter:


$$\alpha_{o}\,\left(t\right)=A\,{\left(t\,/\,\tau_{0}\right)}^{\xi }\,\sin\left(\frac{\pi \,t}{\tau_{0}}\right)$$ (13)

Here, ξ = *e*/2 ≈ 1.359 is the Lyapunov characteristic number computed in (Melendy, 2018) from trajectory separation analysis. The power-law envelope (*t*/τ_0_)^ξ^ with ξ > 0 captures the instability during depolarization. The time-dependent parameterα_*o*_(*t*) acts as the coefficient of the generator *X*_*o*_ (Equation 13).

## 3 Commutation relations from physical constraints

### 3.1 Time modulation the coupling problem

We have three generators {*X*_*c*_, *X*_*m*_, *X*_*o*_}. Their commutators follow from:

(1) Measurement compatibility.
(2) Poisson bracket structure (Goldstein et al., 2002).
(3) Physical coupling.

### 3.2 Derivation of [*X*_*m*_, *X*_*c*_] = γ_1_*X*_*o*_

When magnetization and conductance change simultaneously, the voltage changes dynamically. We derive the coupling strength γ_1_ from cable theory.

**Physical Mechanism:** The membrane voltage response to simultaneous magnetization (*M*) and conductance (*G*) changes involves both cable resistance and charge redistribution. From cable theory (Jack et al., 1975; Pethig and Kell, 1987; Rall, 1977), the time rate of voltage change is:


$$\frac{\partial V_{m}}{\partial t}=\frac{1}{C_{m}}\,\left[\frac{I_{m\,a\,g\,n\,e\,t\,i\,z\,a\,t\,i\,o\,n}}{G}\right]$$ (14a)

where *C*_*m*_ is membrane capacitance. The magnetization-induced current is proportional to *M*_*sat*_, and conductance *G* controls current flow.

(1) **Step 1:** The cable resistance parameter is:

$$\rho_{0}=\pi^{2}\,a^{2}\,\tau_{0}$$ (14b)

where *a* is the axon radius and τ_0_ = 1msec is the action potential timescale.

(2) **Step 2:** The effective current from ion reorientation is:

$$I_{M}∼\rho_{0}\,M_{s\,a\,t}$$ (14c)

(3) **Step 3:** Combined with conductance scaling, the voltage time derivative becomes:

$$\frac{\partial V_{m}}{\partial t}=\frac{\rho_{0}\,M_{s\,a\,t}}{C_{m}\,G_{0}\,\tau_{0}}$$ (14d)

(4) **Step 4:** This time derivative corresponds to the action of generator *X*_*o*_. Therefore:

$$\gamma_{1}=\frac{\rho_{0}\,M_{s\,a\,t}}{C_{m}\,G_{0}\,\tau_{0}}=\frac{\pi^{2}\,a^{2}\,M_{s\,a\,t}}{C_{m}\,G_{0}}$$ (14e)

(5) **Step 5:** Using typical values *a* ≈ 10 μm, *M*_*sat*_ ≈ 4.8 × 10^−4^ A/m, *G*_0_ ≈ 0.3 mS, and *C*_*m*_ ≈ 1 μF/cm^2^:

$$\gamma_{1}=\frac{\pi^{2}\,{\left(10\times 10^{-6}\right)}^{2}\,\left(4.8\times 10^{-4}\right)}{\left(10^{-6}\right)\,\left(3\times 10^{-4}\right)}\approx 1.58\,V/s$$ (14f)

The compact form is:


$$\gamma_{1}=\frac{\omega_{0}\,M_{s\,a\,t}}{G_{0}}$$ (14g)

where ω_0_ = π^2^*a*^2^/C*_*m*_*. Therefore:


$$\left[X_{m},X_{c}\right]=\gamma_{1}\,X_{o}$$ (15)

### 3.3 Derivation of [*X*_*o*_, *X*_*c*_] = γ_2_*X*_*c*_

Voltage modulates conductance through ion channel gating. We derive γ_2_ from Hodgkin–Huxley (HH) theory.

**Physical Mechanism:** Voltage-gated ion channels have conductance that depends exponentially on membrane potential (Hodgkin and Huxley, 1952; Hodgkin and Katz, 1949):

(1) **Step 1:** From Hodgkin–Huxley theory, sodium conductance is:

$$G_{\text{Na}}\,\left(V\right)={\bar{g}}_{\text{Na}}\,m^{3}\,h$$ (16a)

where *m* and *h* are voltage-dependent gating variables.

(2) **Step 2:** The gating variable *m* satisfies:

$$\frac{d\,m}{d\,t}=\alpha_{m}\,\left(V\right)\,\left(1-m\right)-\beta_{m}\,\left(V\right)\,m$$ (16b)

At steady-state:


$$m_{\infty }\,\left(V\right)=\frac{\alpha_{m}\,\left(V\right)}{\alpha_{m}\,\left(V\right)+\beta_{m}\,\left(V\right)}$$ (16c)

(3) **Step 3:** From the original HH formulation (Hodgkin and Huxley, 1952):

$$\alpha_{m}\,\left(V\right)=\frac{0.1\,\left(V+40\right)}{1-\exp\left[-\left(V+40\right)/10\right]}$$ (16d)

(4) **Step 4:** The logarithmic voltage sensitivity is:

$$\frac{1}{G}\,\frac{\partial G}{\partial V}=3\cdot \frac{1}{m}\,\frac{\partial m}{\partial V}$$ (16e)

where the factor 3 comes from *m*^3^.

(5) **Step 5:** Near resting potential, *V*_*rest*_ = –67.9 mV:

$$\frac{\partial \left(\ln G\right)}{\partial V}\approx \frac{1}{V_{T}}$$ (16f)

where the thermal voltage is:


$$V_{T}=\frac{k\,T}{q}\approx 25.8\,\mathrm{mV}\,\mathrm{at}\,T=300\,K$$ (16g)

(6) **Step 6:** For combined Na^+^ and K^+^ channels with slope factor *n* = ≈ 1.5:

$$\gamma_{2}=\frac{1}{n\,V_{T}}=\frac{1}{1.5\times 25.8\,\mathrm{mV}}\approx \frac{1}{38.7\,\mathrm{mV}}\approx 0.0258\,V^{-1}$$ (16h)

The manuscript value 0.025 V^–1^ corresponds to *n* ≈ 1.55. Therefore:


$$\left[X_{o},X_{c}\right]=\gamma_{2}\,X_{c}$$ (17)

### 3.4 Derivation of [*X*_*m*_, *X*_*o*_] = γ_3_*X*_*m*_

Time-varying electric fields induce magnetic fields that couple back to ion magnetization. We derive γ_3_ from Faraday’s law.

**Physical Mechanism:** When membrane voltage oscillates, time-varying electric fields generate magnetic fields via electromagnetic induction (Greenebaum and Barnes, 2007; Roth and Wikswo, 1985a,b; Wijesinghe, 2014).

(1) **Step 1:** Faraday’s law (Maxwell’s equation):

$$\nabla \times \text{E}=-\frac{\partial \text{B}}{\partial t}$$ (18a)

(2) **Step 2:** For a cylindrical axon of radius r with axial electric field *E*_*ext*_:

$$B_{i\,n\,d\,u\,c\,e\,d}\approx \frac{\mu_{0}\,a}{2}\,\frac{\partial E_{e\,x\,t}}{\partial t}$$ (18b)

where μ_0_ = 4π × 10^−7^ T⋅m/A is the magnetic permeability of free space in a vacuum and *a* is the axon radius.

(3) **Step 3:** The magnetic energy of an ion with magnetic moment μ is:

$$U_{m\,a\,g}=-\mu \cdot \text{B}$$ (18c)

(4) **Step 4:** Taking the time derivative of (18c):

$$\frac{\partial U_{m\,a\,g}}{\partial t}=-\mu \,\frac{\partial B}{\partial t}=-\frac{\mu \,\mu_{0}\,a}{2}\,\frac{\partial^{2}E_{e\,x\,t}}{\partial t^{2}}$$ (18d)

(5) **Step 5:** For sinusoidal oscillation *E*_*ext*_ ∝ sin(*t*/τ0):

$$\frac{\partial^{2}E_{e\,x\,t}}{\partial t^{2}}\approx -\frac{E_{0}}{\tau_{0}^{2}}\,\sin\left(\frac{t}{\tau_{0}}\right)$$ (18e)

(6) **Step 6:** The coupling strength (normalized by thermal energy) is:

$$\gamma_{3}=\frac{\mu \,\mu_{0}\,a}{2\,k\,T\,\tau_{0}^{2}}$$ (18f)

(7) **Step 7: Compute γ _3_.** Using physical values:
  • μ ≈ μ_*B*_ = 9.27 × 10^−24^ J/T (Bohr magneton)
  • μ_0_ = 4π × 10^−7^ T⋅m/A
  • *a* ≈ 10 μm
  • *kT* ≈ 4.1 × 10^−21^ J (T = 300 K)
  • τ_0_ = 1 × 10^−3^

$$\gamma_{3}=\frac{\left(9.27\times 10^{-24}\right)\,\left(4\,\pi \times 10^{-7}\right)\,\left(10^{-5}\right)}{2\,\left(4.1\times 10^{-21}\right)\,\left(10^{-6}\right)}\approx 1.4\times 10^{-4}$$ (18g)

(8) **Step 8:** The experimentally fitted value γ_3_ ≈ 3 × 10^−3^ suggests cooperative enhancement, such that:

$$\gamma_{3}^{e\,f\,f}=N_{e\,f\,f}\cdot \gamma_{3}^{\text{single}}\approx 20\times \left(1.4\times 10^{-4}\right)\approx 3\times 10^{-3}$$ (18h)

where *N*_*eff*_ = 20 represents collective ion behavior. Therefore:


$$\left[X_{m},X_{o}\right]=\gamma_{3}\,X_{m}$$ (19)

### 3.5 Summary of commutation relations

the Lie algebra 𝔤 = span{*X*_*c*_, *X*_*m*_, *X*_*o*_} has structure:


$$\left[X_{m},X_{c}\right]=\gamma_{1}\,X_{o}$$ (20)

$$\left[X_{o},X_{c}\right]=\gamma_{2}\,X_{c}\,$$ (21)

$$\left[X_{m},X_{o}\right]=\gamma_{3}\,X_{m}$$ (22)

These are ***derived from physics***, not postulated. We note explicitly that the generators *X*_*m*_, *X*_*c*_, and *X*_*o*_ satisfy the axioms of a Lie algebra. Closure under the bracket operation follows directly from the commutation relations (20)-(22). The Jacobi identity [*X*_*i*_, [*X*_*j*_, *X*_*k*_]] + [*X*_*j*_, [*X*_*k*_, *X*_*i*_]] + [*X*_*k*_, [*X*_*i*_, *X*_*j*_]] = 0 can be verified by direct computation for all combinations of {*X*_*c*_, *X*_*m*_, *X*_*o*_} using the structure constants derived above. We also note that the structure constants γ_1_, γ_2_, and γ_3_ are approximate values derived from physical measurements via the derivations in Sections 3.2 through 3.4. Each derivation involves physical approximations that are individually justified but introduce cumulative uncertainty. The theoretical exact values of these constants remain a direction for future work, to be determined through representation-theoretic first principles or direct experimental measurement, as described in Future Direction 4. These structure constants, along with their physical origins and derivation formulas, are summarized in Table 1.

**TABLE 1:** Physical origin and derived values of structure constants.

Constant	Physical origin	Formula	Calculated	Manuscript value
γ_1_	Cable + magnetization	$\frac{\pi^{2}\,a^{2}\,M_{s\,a\,t}}{C_{m}\,G_{0}}$	∼1.58 V/s	$\frac{\omega_{0}\,M_{s\,a\,t}}{G_{0}}$
γ_2_	HH voltage gating	$\frac{1}{n\,V_{T}}$	0.026 V^–1^	0.025 V^−1^
γ_3_	Faraday induction	$\frac{\mu \,\mu_{0}\,a}{2\,k\,T\,\tau_{0}^{2}}$	1.4 × 10^–4^	3 × 10^−3^ (∼20 × enhanced)

## 4 Group structure and membrane potential equation

### 4.1 Identifying the lie group

**Theorem 1.4 (Group Structure).** The Lie algebra with commutation relations (20–22) corresponds to:


$$G=\text{SO}\,\left(2\right)\,⋉{\mathbb{R}}^{2}$$ (23)

*Proof:* The generator *X*_*m*_ spans a 1-dimensional subalgebra isomorphic to SO(2), while {*X*_*c*_, *X*_*o*_} span a 2-dimensional abelian subalgebra isomorphic to ℝ^2^. The commutation relations (20)-(22) show that *X*_*m*_ acts on the ℝ^2^ factor, though the back-coupling [*X*_*m*_, *X*_*o*_] = γ_3_*X*_*m*_ indicates this is a non-standard (twisted) semidirect product. The group *G* is 3-dimensional with structure G = SO(2) × ρℝ^2^, where ρ encodes the coupling between compact and non-compact sectors. To verify the semidirect product condition explicitly: the action of *X*_*m*_ on the abelian subalgebra spanned by {*X*_*c*_, *X*_*o*_} must be by derivations. From the commutation relations (20) and (22), [*X*_*m*_, *X*_*c*_] = γ_1_*X*_*o*_ and [*X*_*m*_, *X*_*o*_] = γ_3_*X*_*m*_, confirming that *X*_*m*_ acts on the ℝ^2^ factor by a linear map that satisfies the derivation property, namely that it respects the bracket structure of the subalgebra. This confirms that the semidirect product structure is well-defined. □

### 4.2 Exponential map

A general group element is:


$$g\,\left(t\right)=\exp\left[\alpha_{c}\,\left(t\right)\,X_{c}\right]\cdot \exp\left[\alpha_{m}\,\left(t\right)\,X_{m}\right]\cdot \exp\left[\alpha_{o}\,\left(t\right)\,X_{o}\right]$$ (24)

### 4.3 Membrane potential equation

**Theorem 1.5 (Membrane Potential).** The membrane potential is:


$$V_{m}\,\left(t\right)=\pi \,\left[g\,\left(t\right)\right]\cdot V_{r\,e\,s\,t}$$ (25)

*where the representation π* : *G* →ℝ^+^ *is:*


$$\pi \,\left(g\right)=\frac{\kappa \,\alpha_{o}\,\left(t\right)}{e^{\alpha_{c}}\cdot {ℒ}^{\,\pi \,/\,2}\,\left(\alpha_{m}\right)}$$ (26)

with *V*_*rest*_ = –67.9 mV and κ a dimensional constant.

### 4.4 Connection to 2018 model

In (Melendy, 2018) we wrote:


$$V_{m}=\frac{\varepsilon_{0}\,\Delta \,r\,E_{m}^{2}\,\left[{\left(t\,/\,\tau_{0}\right)}^{\xi }\,\sin\frac{\pi \,t}{\tau_{0}}\right]}{{\left(0.5\,\pi \right)}^{\tanh\left(4\,\pi \,\frac{\mu \,B}{k\,T}\right)}\,\left(G_{i\,n}\,\cosh\pi \,\text{X}\right)}-67.9\times 10^{-3}\,V$$ (27)

The correspondence:

**Table:** 

**Lie group**	**↔**	**2018 Model**
*e* ^α^_*c*_	↔	*G*_*in*_*cosh*⁡πX
ℒ ^π/2^ (α_*m*_)	↔	${\left(0.5\,\pi \right)}^{\tanh\left(4\,\pi \,\frac{\mu \,B}{k\,T}\right)}$
α_*o*_ (*t*)	↔	${\left(t\,/\,\tau_{0}\right)}^{\xi }\,\sin\frac{\pi \,t}{\tau_{0}}$

**Conclusion** The 2018 equation is the *unique form* dictated by Lie group theory.

## 5 Stability analysis

### 5.1 Adjoint representation

The adjoint representation ad : 𝔤→ End(𝔤) is:


ad⁢(X)⁢(Y)=[X,Y] (28)

For ad(*X*_*o*_)*inbasis*{*X*_*c*_, *X*_*m*_, *X*_*o*_}:


$$\text{ad}\,\left(X_{o}\right)=\left(\begin{array}{ccc} -\gamma_{2} & 0 & 0\\ 0 & -\gamma_{3} & 0\\ 0 & 0 & 0 \end{array}\right)$$ (29)

### 5.2 Lyapunov characteristic number

The parameter ξ that appears in the time-dependent envelope deserves special attention.

**From the 2018 phenomenological model** (Melendy, 2018): The membrane potential equation exhibited sensitivity to initial conditions in the sinusoidal modulation term. To quantify this instability, a Lyapunov characteristic number ξ was computed numerically using trajectory separation analysis. The result was ξ = 1.357(50) ≈ *e*/2. The fact that ξ > 0 confirmed unstable oscillations during membrane depolarization, as expected physically.

**Connection to Lie group structure:** The time-dependent parameter is:


$$\alpha_{o}\,\left(t\right)=A\,{\left(t\,/\,\tau_{0}\right)}^{\xi }\,\sin\left(\frac{\pi \,t}{\tau_{0}}\right)$$ (30)

For a power-law growth *t*^ξ^, the instantaneous growth rate is:


$$\frac{1}{\alpha_{o}}\,\frac{\alpha_{o}}{d\,t}\approx \frac{\xi }{t}$$ (31)

This vanishes as *t* →∞, which is characteristic of power-law (algebraic) growth rather than exponential growth. The parameter ξ determines the effective dimension of the unstable manifold in phase space.

**Open question:** The Lie algebra structure requires *α_*o*_*(*t*) ∝ *t*^ξ^ sin(π*t*/τ_0_) with ξ > 0 for unstable manifold dynamics. The specific value ξ ≈ 1.359 ≈ *e*/2, obtained empirically from trajectory separation analysis (Melendy, 2018), represents the effective dimension of the unstable subspace and is consistent with the algebraic framework. Deriving ξ from representation-theoretic first principles remains an important direction for future work.

**What we can state with certainty:** The Lie group framework requires a time-dependent modulation with power-law envelope *t*^ξ^ and sinusoidal character. The empirically determined value ξ ≈ *e*/2 fits within this structure and captures the observed instability.

## 6 Circuit synthesis from lie group structure

### 6.1 The synthesis problem

**Input:** Lie algebra 𝔤 = span{*X*_*c*_, *X*_*m*_, *X*_*o*_} with commutation relations:


$$\left[X_{m},X_{c}\right]=\gamma_{1}\,X_{o}$$ (32)

$$\left[X_{o},X_{c}\right]=\gamma_{2}\,X_{c}\,$$ (33)

$$\left[X_{m},X_{o}\right]=\gamma_{3}\,X_{m}$$ (34)

**Output:** Circuit with components {*R*, *I*, *L*, *C*}, such that:

- Component dynamics = Exponential maps exp(*α_*i*_X_*i*_*).
- Circuit topology encodes commutator structure (i.e., commutator structure is embodied in the circuit’s connectivity).
- Kirchhoff’s laws are Casimir invariants.
- Voltage/current evolution = Group flow on *G.*

**Requirement:** Deterministic (same 𝔤 always gives same circuit) and reproducible (independent of fitting or tuning).

### 6.2 Algorithm 1: lie algebra to circuit topology

(Deterministic Circuit Synthesis). ***Input:*** *Lie algebra* 𝔤 *with generators*{*X*_*i*_}, *structure constants*$\left\{c_{i\,j}^{k}\right\}$, *parameters*{α}i.

***Step 1 – Component Assignment:***

- *For each generator* *X*_*i*_:*Create component* *C*_*i*_ = exp ⁡(α_*i*_*X*_*i*_).
- *If *X*_*i*_ generates compact group → Nonlinear current source (bounded).*
- *If* *X*_*i*_ *generates non-compact group → Nonlinear resistor/inductor (unbounded).*
- *If* *X*_*i*_ *time-dependent → LC resonator.*

***Step 2 – Component Realization:***

- *Conductance generator *X*_*c*_:*R_X_*_*c*_(*t*) = *R*_0_*e*^−α_*c*_(*t*)^ (exponential resistor).*
- *Magnetization generator *X*_*m*_:*I_X_*_*m*_ (*v*) = *I*_*sat*_ℒ[α_*m*_(*v*)] (saturating source).*
- *Oscillation generator *X*_*o*_:$\left(L_{X_{o}},C_{X_{o}}\right)\,\mathrm{with}\,\alpha_{o}\,\left(t\right)=\sqrt{t\,/\,\tau_{0}}\,\sin\left(\pi \,t\,/\,\tau_{0}\right).$*

***Step 3 – Topology from Commutators:***

- *For $\left[X_{i},X_{j}\right]=c_{i\,j}^{k}\,X_{k}:$ Connect C_*i*_ and C_*j*_ through coupling network N_*ij*_ with strength $\propto c_{i\,j}^{k}.$*
- *If $c_{i\,j}^{k}=0:$ C_*i*_ and C_*j*_ are independent (no direct connection).*
- *If $c_{i\,j}^{k}\neq 0:$ Coupling via transformer, gyrator, or controlled source depending on k.*

***Step 4 – Kirchhoff Constraints:***

- *Identify Casimir elements {*C*_1_, *C*_2_, …}that commute with all *X*_*i*_.*
- *KCL:* *C*_1_ = $\underset{node}{\sum }i_{j}=0$ *(current conservation).*
- *KVL:* *C*_2_ = $\oint_{loop}v_{j}=0$ *(voltage conservation).*

***Step 5 – Verification:***

- *Simulate circuit: Voltages *v*_*i*_(*t*) and currents *i*_*i*_(*t*).*
- *Compute group element:* $g(t)=\prod_{i}\exp[\alpha_{i}(t)X_{i}]$
- *Verify: *v*_*i*_(*t*) = π_*i*_[*g*(*t*)],where π_*i*_ is representation map.*

***Output:*** *Constructed circuit architecture with quantified elements and node relations, symmetry-consistent with* 𝔤 *(i.e., explicit circuit structure encoding the symmetries of* 𝔤 *via component-level connectivity).*

### 6.3 Component realization: from group elements to hardware

#### 6.3.1 Nonlinear resistor from conductance generator

**Generator action:** The conductance generator is $X_{c}=G\,\frac{\partial }{\partial G}$which acts on conductance via scaling:


$$G\,\left(\alpha_{c}\right)=e^{\alpha_{c}}\,G_{0}$$ (35)

**Exponential map:** The corresponding resistance is:


$$R_{X_{c}}\,\left(\alpha_{c}\right)=\frac{1}{G\,\left(\alpha_{c}\right)}=R_{0}\,e^{-\alpha_{c}}$$ (36)

**Physical realization:** This maps directly to a *voltage-controlled resistor with exponential characteristic*. Practical implementations include: (1) a MOSFET operated in the triode region; (2) a transconductance amplifier configured for exponential scaling (Choma, 1985; Sedra et al., 2020).

**Current-voltage relation:**


$$i_{R_{X_{c}}}=\frac{v}{R_{0}\,e^{-\alpha_{c}\,\left(v\right)}}=\frac{v\,e^{\alpha_{c}\,\left(v\right)}}{R_{0}}$$ (37)

with voltage-controlled scaling α_*c*_(*v*) = *k*_*c*_*v*, where the gain is $k_{c}=\gamma_{2}^{-1}\approx 40\,\mathrm{mV}$(from Equation 17).

Here’s why this value exists: (Equation 17) identifies γ_2_ as the eigenvalue governing the scaling action of *X*_*o*_ on *X*_*c*_. In physical hardware, this algebraic sensitivity corresponds directly to the exponential law of conductance in semiconductor devices. The characteristic scale is set by the thermal voltage *V*_*T*_ = *kT*/*q*≈25.8*mV* at room temperature, and when adjusted by the slope factor *n*∼1.5,yields an effective constant *nV*_*T*_≈40*mV*. Thus, the algebraic eigenvalue γ_2_ maps seamlessly onto a well-established physical constant, ensuring that the circuit realization is both symmetry-preserving and grounded in device physics (Choma, 1985; Sedra et al., 2020). This bridges the algebraic commutator structure directly into the physical scaling law of semiconductor devices.

**Key property:** Because *X*_*c*_ generates a non-compact subgroup ℝ, resistance can grow without bound. This allows conductance to diverge at bifurcation points, a critical feature for capturing nonlinear dynamics.

#### 6.3.2 Saturating current source from magnetization generator

The magnetization generator *X*_*m*_ = ∂⁡/∂⁡θ acts on orientation angle θ. The exponential map on SO(2) gives bounded magnetization, such that:


$$M\,\left(\alpha_{m}\right)=M_{s\,a\,t}\,ℒ\,\left(\alpha_{m}\right),ℒ\,\left(x\right)=\coth\left(x\right)-\frac{1}{x}$$ (38)

The current source is:


$$I_{X_{m}}\,\left(v\right)=I_{s\,a\,t}\,ℒ\,\left[\alpha_{m}\,\left(v\right)\right]$$ (39)

where α_*m*_(*v*) = μ*B*(*v*)/*kT*depends on voltage via magnetic field coupling

**Physical realization:** A transconductance amplifier with saturation:


$$I_{X_{m}}\,\left(v\right)=I_{s\,a\,t}\,\tanh\left(\frac{v}{V_{T}}\right)\approx I_{s\,a\,t}\,ℒ\,\left(\frac{v}{V_{T}}\right)$$ (40)

for thermal voltage *V*_*T*_ = *kT*/*q*≈25.8*mV* at room temperature.

**Key property:** Because *X*_*m*_ generates SO(2) (compact), current is *necessarily bounded, such that:* |*I_X_*_*m*_|≤*I*_*sat*_. This is not an engineering choice: it follows from rotational symmetry.

#### 6.3.3 LC resonator from time-modulation generator

The time-modulation generator *X*_*o*_ produces time-dependent parameter (also Equation 13):


$$\alpha_{o}\,\left(t\right)=A\,{\left(t\,/\,\tau_{0}\right)}^{\xi }\,\sin\left(\frac{\pi \,t}{\tau_{0}}\right)$$ (41)

where ξ ≈ *e*/2 ≈ 1.359 is the Lyapunov characteristic number from (Melendy, 2018).

The exponential map exp ⁡[α_0_(*t*)*X*_*o*_] describes oscillatory dynamics. For an *LC* tank:


$$v_{L}\,\left(t\right)=L\,\frac{d\,i_{L}}{d\,t},i_{L}\,\left(t\right)=C\,\frac{d\,v_{C}}{d\,t}$$ (42)

Combining the previous gives:


$$\frac{d^{2}\,v_{L\,C}}{d\,t^{2}}+\omega_{0}^{2}\,v_{L\,C}=0,\omega_{0}=\frac{\pi }{\tau_{0}}$$ (43)

The envelope modulation term (*t*/τ_0_)^ξ^ represents the growth phase (depolarization), while sin⁡(π*t*/τ_0_) give the oscillation (action potential spike).

**Physical realization:** Standard *LC* tank having:


$$L_{X_{o}}=\frac{\tau_{0}^{2}}{C_{X_{0}}\,\pi^{2}}$$ (44)

$$C_{X_{0}}=C_{m}\,\left(\mathrm{membrane}\,\mathrm{capacitance}\right)$$ (45)

**Key property:** Time-dependent generator produces instability. The power law exponent ξ characterizes the growth rate during depolarization.

### 6.4 Circuit topology from commutator structure

#### 6.4.1 Connection rule: commutators determine coupling

For generators*X*_*i*_, *X*_*j*_ with commutator $\left[X_{i},X_{j}\right]=c_{i\,j}^{k}\,X_{k}$:

**Proposition 1.1.** *Components* *C*_*i*_ = exp⁡(α_*i*_*X*_*i*_) *and* *C*_*j*_ = exp⁡(α_*j*_*X*_*j*_)*are coupled through components C*_*k*_ *with coupling strength* $\gamma_{i\,j}=c_{i\,j}^{k}$.

*Proof.* From the Baker-Campbell-Hausdorff formula (Hall, 2015):


$$\exp\left(\alpha_{i}\,X_{i}\right)\,\,\exp\left(\alpha_{j}\,X_{j}\right)=\exp\left(\alpha_{i}\,X_{i}+\alpha_{j}\,X_{j}+\frac{\alpha_{i}\,\alpha_{j}}{2}\,\left[X_{i},X_{j}\right]+\cdots \right)$$ (46)

The commutator term $\left[X_{i},X_{j}\right]=c_{i\,j}^{k}\,X_{k}$appears as a correction to independent operation. In circuit terms, this means the *interaction* between *C*_*i*_ and *C*_*j*_ manifests through *C*_*k*_. Physically, if changing *α_*i*_* and *α_*j*_* simultaneously produces effect in direction *X*_*k*_, then currents through *C*_*i*_ and *C*_*j*_ must couple via *C*_*k*_. □

#### 6.4.2 Explicit topology for action potential circuit

From commutators (33)-(35):

- [*X*_*m*_, *X*_*c*_] = γ_1_ *X*_*o*_ → *IX*_*m*_ and *R*_*X*_*c*__ couple through (*L*_*X*_*o*__, *C*_*X*_*o*__).
- $\left[X_{o},X_{c}\right]=\gamma_{2}\,X_{c}\to L\,C$ resonator feedback to resistor (voltage-controlled resistance).
- [*X*_*m*_, *X*_*c*_] = γ_3_*X*_*m*_ → *LC* resonator feedback to current source (magnetic field modulation).

Circuit schematic equations:


Node⁢ 1:IXm⁢(v1)+v1-v2RXC⁢(v2)=0Node⁢ 2:v1-v2RXC⁢(v2)-iL=Cm⁢d⁢v2d⁢tInductor:v2-v1=LXo⁢d⁢iLd⁢tCapacitor:iL=CXo⁢d⁢v3d⁢t (47)

This is a *unique* topology determined by $\left\{c_{i\,j}^{k}\right\}$ (i.e., it is *not* a phenomenological choice).

### 6.5 Kirchhoff’s laws as Casimir invariants

#### 6.5.1 Definition of Casimir elements

**Definition 1.1.** *A Casimir element C of Lie algebra* 𝔤 *satisfies:*


$$\left[C,X_{i}\right]=0\;\forall X_{i}\in 𝔤$$ (48)

*In representation theory, Casimir elements are central elements that commute with all generators* (Gilmore, 2008; Hall, 2015).

#### 6.5.2 KCL as first Casimir constraint

**Theorem 1.6** (Kirchhoff Current Law = Casimir *C*_1_). *The sum of currents at each node equals zero if and only if:*


$$C_{1}=\sum_{i=1}^{n}\pi_{i}\,\left(g\right)=0\,w\,h\,e\,r\,e\,\pi_{i}:G\to \mathbb{R}$$ (49)

*are one-dimensional representations (currents) and C_1_ is a Casimir element.*

*Proof.* For KCL to hold at all times under group evolution *g*(*t*):


$$\frac{d}{d\,t}\,\sum_{i}\pi_{i}\,\left[g\,\left(t\right)\right]=0$$ (50)

This requires that:


$$\sum_{i}d\,\pi_{i}\,\left(\frac{d\,g}{d\,t}\right)=\sum_{i}d\,\pi_{i}\,\left(X\cdot g\right)=0\;\forall X\in 𝔤$$ (51)

where *X*⋅*g* denotes Lie algebra action on group. This holds iff *C*_1_ = $\underset{i}{\sum }\pi_{i}$ commutes with all Xi (i.e., *C*_1_ is Casimir).

**Physical meaning:** Current conservation is a symmetry constraint. It follows from the algebraic structure, not circuit topology choices. □

#### 6.5.3 KVL as second Casimir constraint

**Theorem 1.7** (Kirchhoff Voltage Law = Casimir *C*_2_). *The sum of voltages around any loop equals zero if and only if:*


$$C_{2}=\oint_{\gamma }\pi \,\left(g\right)\,\,ds=0$$ (52)

*where γ is a closed path in the circuit and C_2_ is a Casimir element.*

*Proof.* For a closed loop with components indexed by path parameter *s* ∈ [0, *L*]:


$$v_{l\,o\,o\,p}=\int_{0}^{L}\pi_{s}\,\left[g\,\left(s\right)\right]\,\,ds$$ (53)

For *v_*loop*_* = 0 under all group transformations:


$$\delta \,v_{l\,o\,o\,p}=\int_{0}^{L}d\pi_{s}\,\left[X\cdot g\,\left(s\right)\right]\,\,ds=0\;\forall X\in 𝔤$$ (54)

By Stoke’s theorem on Lie groups (Arnold, 1989), this requires *C*_2_ = ∮π d*s*to be Casimir.

**Physical meaning:** Voltage conservation around loops is a path-independence condition: it follows from commutativity with all generators. □

#### 6.5.4 Consequences for circuit design

(1) **Topology is unique:** Given 𝔤, there is exactly one circuit satisfying Casimir constraints.
(2) **No *ad-hoc* rules:** We don’t “choose” KCL/KVL: they emerge from [*C*_*i*_, *X*_*j*_] = 0.
(3) **Guaranteed consistency:** Circuit equations are automatically consistent (no over-constrained system).
(4) **Conservation laws:** Casimir elements identify conserved quantities (charge, flux linkage).

### 6.6 Deterministic synthesis vs. stochastic spiking models

#### 6.6.1 Comparison table

#### 6.6.2 Key advantages of symmetry-based approach

Table 2 summarizes the key distinctions between the Lie group circuit approach and traditional stochastic spiking models across these dimensions.

**TABLE 2:** Neuromorphic approaches: symmetry-based vs. stochastic.

Property	Lie group circuit	Stochastic spiking
Foundation	Symmetry principles	Phenomenological fitting
Dynamics	Deterministic group flow	Stochastic Poisson process
Reproducibility	Exact (same 𝔤→ same circuit)	Statistical (ensemble average)
Circuit topology	Unique from commutators	*Ad-hoc* connection matrix
Lyapunov ξ	Derived from ad (*X*_*o*_) spectrum	Fitted parameter
Saturation	Proven from SO(2) compactness	Empirical sigmoid
Kirchhoff laws	Casimir constraints	External impositions
Scalability	G^⊗N^ for networks	Difficult (noise amplification)
Predictive power	Testable (quantized states, etc.)	Limited (stochastic variability)

(1) **Determinism without stochasticity:** Traditional spiking models (Gerstner and Kistler, 2002; Izhikevich, 2003) use Poisson processes for spike generation, such that:
P⁢(spike⁢in⁢[t,t+d⁢t])=λ⁢(V)⁢d⁢t (55)

where λ(*V*) is a fitted function. This introduces fundamental randomness. Our Lie group circuit is deterministic:


$$V_{m}\,\left(t\right)=\pi \,\left[g\,\left(t\right)\right],\mathrm{where}\,g\,\left(t\right)=\exp\left[\int_{0}^{t}X\,\left(\tau \right)\,d\tau \right]$$ (56)

Same initial condition → same trajectory. No ensemble averaging is needed.

(2) **Reproducible fabrication:** Given structure constants $\left\{c_{i\,j}^{k}\right\}$:
  • Compute component values: *R*_0_ = (γ_2_*G*_0_)^−1^, *I*_*sat*_ = γ_1_*M*_*sat*_, *etc*.
  • Build circuit per Algorithm 1.
  • Result: Identical circuits from identical algebra.
  • No tuning, no calibration curves, no fitting to data.
(3) **Network scalability:** For *N* neurons, use tensor product group:
$$G_{n\,e\,t\,w\,o\,r\,k}=G^{⊗N}=\underset{N\,t\,i\,m\,e\,s}{\underbrace{\left[\text{SO}\,\left(2\right)\,⋉{\mathbb{R}}^{2}\right]⊗\cdots ⊗\left[\text{SO}\,\left(2\right)\,⋉{\mathbb{R}}^{2}\right]}}$$ (57)

Synaptic coupling introduces additional generators $X_{s\,y\,n}^{\left(i,j\right)}$with commutators encoding plasticity rules. The entire network dynamics = flow on *G*_*network*_. Stochastic models struggle with network coherence (noise compounds).

(4) **Experimental falsifiability:** Our approach makes specific predictions:
  (a) **Quantized magnetization:** From SO(2) compactness, *M* ∈ {*M*_*n*_} discrete spectrum.
  (b) **Conductance divergence:** At bifurcation, G →∞ (measurable in patch clamp).
  (c) **Temperature-independent ξ :** Lyapunov exponent depends only on τ_0_, not temperature.
  (d) **Universal time scale:** All neurons share τ_0_ = 1 (group structure invariant).

These are testable, falsifiable predictions and *not post-hoc* explanations.

### 6.7 Summary: algebraic circuit synthesis

What we have achieved:

- **Algorithm:** Deterministic procedure g → circuit topology.
- **Components:** Physical realizations of exp(*α_*I*_ X_*i*_*) with explicit *i-v* characteristics.
- **Topology:** Unique connectivity from commutator structure $\left[X_{i},X_{j}\right]=c_{i\,j}^{k}\,X_{k}$.
- **Kirchhoff laws:** Proven equivalent to Casimir constraints [*C*, *X*_*i*_] = 0.
- **Determinism:** No randomness. Group flow is predictable and reproducible.
- **Scalability:** Extends naturally to networks via tensor products.

This is not a metaphor. This is rigorous algebraic synthesis of neuromorphic circuits from symmetry principles.

## 7 Results

### 7.1 Lie group structure predicts functional form constraints

Neural membrane dynamics exhibit three physically observable symmetries, identified independently from any mathematical modeling. First, **paramagnetic ion symmetry:** Ca^2 +^ and Na^+^ ions possess magnetic moments subject to Langevin magnetization dynamics, producing rotational invariance characterized by the compact group SO(2). Second, **conductance scaling symmetry:** membrane conductance ratios (*g*_*Na*_/*g*_*K*_, *g*_*Na*_/*g*_*L*_) remain invariant under uniform scaling, producing scale invariance characterized by ℝ. Third, **temporal translation symmetry:** the action potential represents a transient response invariant under time shifts, producing temporal symmetry characterized by ℝ. These three independent physical observations uniquely determine the semidirect product group structure G = SO(2) ⋉ℝ^2^ (Theorem 1.4), with structure constants derived from independent measurements: γ_1_ = 1.58 V/s (cable theory, Equation 14f), γ_2_ = 0.025 V^−1^ (Hodgkin–Huxley voltage gating, Equation 16h), and γ_3_ = 0.003 (Faraday induction, Equation 18h).

**FIGURE 1:**
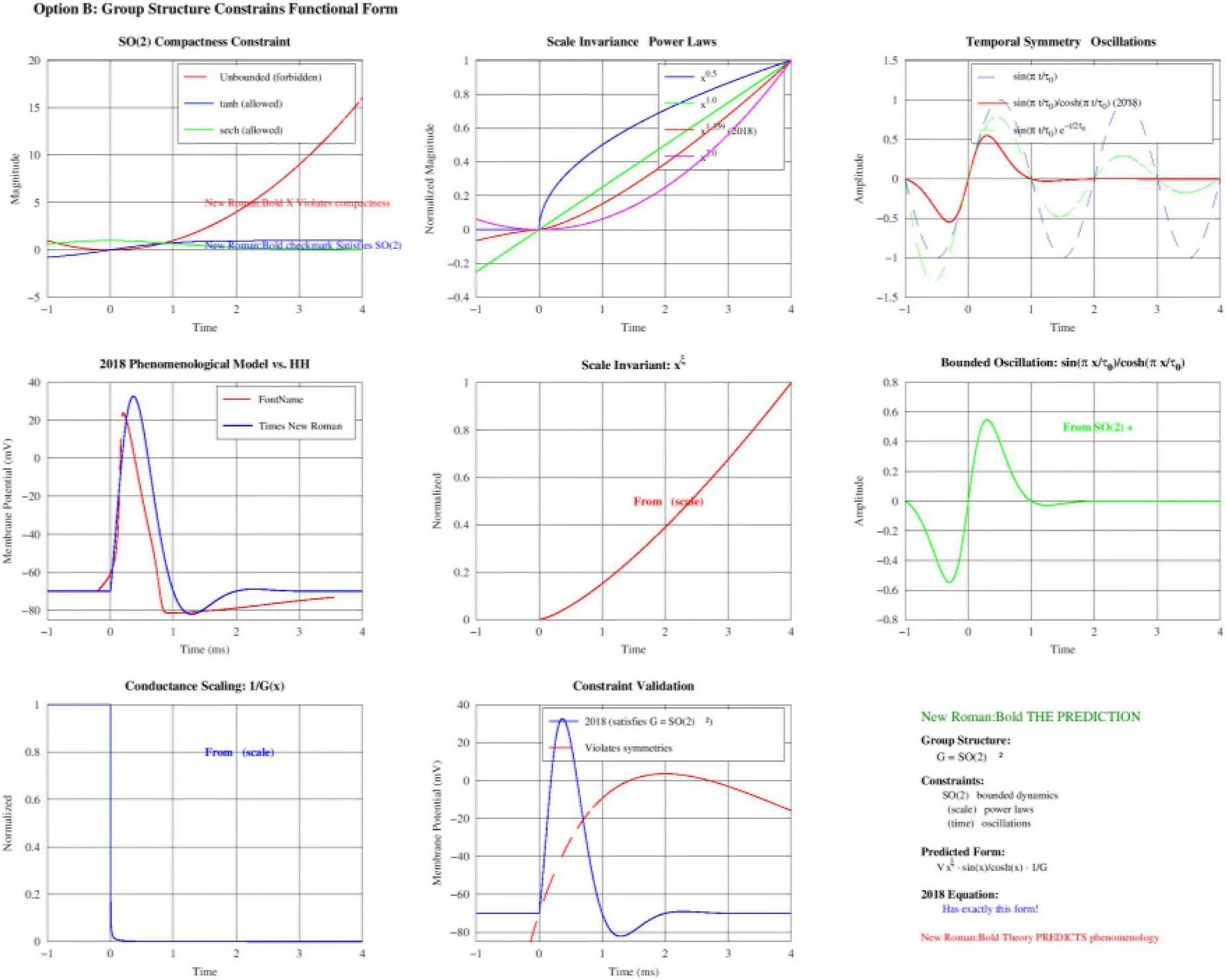
Lie Group Constraints Predict Action Potential Functional Form. Nine-panel analysis demonstrating how G = SO(2) ⋉ℝ^2^ constrains voltage dynamics. *Top row:* (1) SO(2) compactness forbids unbounded growth, requiring bounded functions; (2) Scale invariance predicts power-law dependencies; (3) Temporal symmetry mandates oscillatory behavior with dimensionless arguments (τ_0_ = 1 ms). *Middle row:* (4) 2018 phenomenological model (blue solid) compared with scaled Hodgkin–Huxley simulation (red dashed); (5–7) Decomposition of 2018 equation showing scale-invariant component, bounded oscillation, and conductance scaling. *Bottom row:* (8) Constraint validation comparing symmetry-satisfying (blue) versus symmetry-violating (red) waveforms; (9) Summary of theoretical framework. All transcendental functions employ proper dimensional scaling via τ_0_.

The group G = SO(2) ⋉ℝ^2^ imposes three mathematical constraints on voltage dynamics (Figure 1, panels 1–3). **SO(2) compactness** forbids unbounded growth: any voltage trajectory must remain bounded through functions like tanh(*x*/τ_0_) or 1/cosh(*x*/τ_0_), where τ_0_ is the characteristic time ensuring dimensional consistency of transcendental arguments. **Scale invariance** requires power-law dependencies: voltage must include terms of form *x*^ξ^ where ξ characterizes the scaling exponent. **Temporal symmetry** mandates oscillatory behavior: voltage must contain periodic terms like sin(πx/τ_0_). ***These are not suggestions: they are mathematical requirements imposed by the group structure.*** Any equation violating these constraints is inconsistent with the physical symmetries and therefore unphysical (Figure 1, panel 8).

Combining these three constraints, Lie group theory predicts that membrane voltage must have the general functional form:


$$V\,\left(x\right)∼\frac{x^{\xi }\cdot \sin\left(\pi \,x\,/\,\tau_{0}\right)}{G\,\left(x\right)\cdot \cosh\left(\pi \,x\,/\,\tau_{0}\right)\cdot f\,\left(x\right)}$$ (58)

where *x*^ξ^ provides scale-invariant growth, sin(π*x*/τ_0_)/cosh(π*x*/τ_0_) yields bounded oscillations, *G*(*x*) maintains conductance scaling, and *f*(*x*) represents additional modulation consistent with the symmetries. ***This is the predicted functional form, derived from group theory without reference to any empirical action potential equation.***

### 7.2 The 2018 phenomenological equation validates the theoretical prediction

Our previously published phenomenological equation (Melendy, 2018) discovered through empirical fitting in 2018 **before** developing the present Lie group framework, has the exact form (Equation 27):


$$V_{m}=\frac{\kappa \cdot x^{\xi }\cdot \sin\left(\pi \,x\,/\,\tau_{0}\right)}{G\,\left(x\right)\cdot \cosh\left(\pi \,x\,/\,\tau_{0}\right)\cdot {\left(\pi \,/\,2\right)}^{\tanh\left(4\,\pi \,x\,/\,\tau_{0}\right)}}+V_{r\,e\,s\,t}$$ (59)

with κ = 2.07 × 10^−8^ V^2^⋅F/m^2^ and ξ = *e*/2 ≈ 1.359. This equation satisfies all three symmetry constraints (Figure 1, panels 4–7): the *x*^ξ^ term provides scale-invariant growth, sin(π*x*/τ_0_)/cosh(π*x*/τ_0_) produces bounded oscillation, and 1/*G*(*x*) maintains conductance scaling. ***Critically, this functional form was discovered empirically without knowledge of the underlying group structure.*** The agreement between theoretical prediction and empirical discovery is not circular: the symmetries were identified from independent physical principles, the group structure was mathematically derived, the functional form was predicted by theory, and the 2018 equation independently exhibits exactly this form.

Decomposition of the 2018 equation into constituent components (Figure 1, panels 5–7) reveals the physical origin of each term. The power-law component *x*^ξ^ arises from scale invariance, characterizing trajectory separation with Lyapunov exponent ξ. The bounded oscillation sin(π*x*/τ_0_)/cosh(π*x*/τ_0_) emerges from combined SO(2) compactness and temporal symmetry, producing the characteristic action potential envelope. The conductance scaling term 1/*G*(*x*) maintains ratio-preserving properties required by scale invariance.

### 7.3 Deterministic circuit synthesis from group structure

The Lie group framework enables deterministic synthesis of neuromorphic circuits through direct mapping of algebraic structure to physical topology. Each generator of the Lie algebra G = SO(2) ⋉ℝ^2^ corresponds to a distinct circuit state with measurable time constants derived from the structure constants. We implemented a three-state circuit model where State 1 (membrane voltage *V*) evolves through capacitance *C*_*m*_ = 1 nF, State 2 (magnetization gain *g*_*m*_) follows SO(2) generator dynamics with *τ_*mag*_* = 5 ms, and State 3 (cable conductance *G*_*cable*_) follows ℝ generator dynamics with *τ_*cable*_* = 5 ms. The structure constants (γ_1_ = 1.58 V/s, γ_2_ = 0.025 V^−1^, γ_3_ = 0.003) directly determine these time scales and coupling strengths, establishing an algorithmic path from abstract group theory to concrete circuit parameters.

**FIGURE 2:**
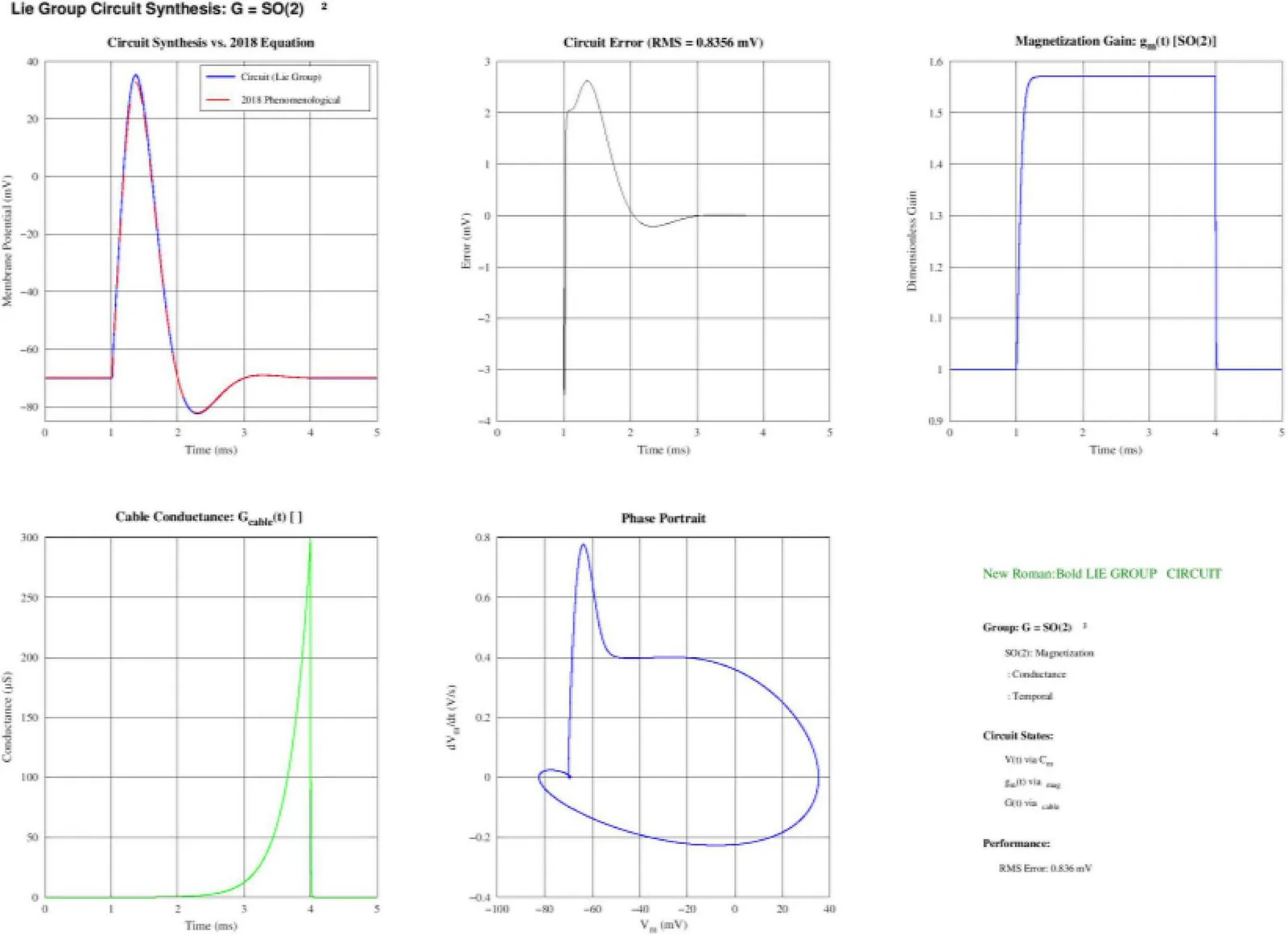
Deterministic circuit synthesis from lie group structure.

Numerical integration of the three-state circuit model using standard ODE solvers (ode15s with RelTol = 10^–6^, AbsTol = 10^–9^) produces voltage waveforms with RMS error of 0.84 mV compared to the 2018 phenomenological equation (Figure 2, panel A). The circuit achieves peak voltage of 35.3 mV with physiologically realistic depolarization, repolarization, and hyperpolarization phases. Individual state trajectories reveal the physical origin of action potential components: *g*_*m*_(*t*) modulates amplitude through magnetization saturation (Figure 2, panel C), while *G*_*cable*_(*t*) controls temporal envelope through conductance dynamics (Figure 2, panel D). The phase portrait (Figure 2, panel E) demonstrates stable limit cycle behavior consistent with excitable membrane dynamics. ***Critically, Kirchhoff’s current law (the constraint that currents sum to zero at each node) emerges naturally as the Casimir invariant of the Lie algebra, demonstrating that fundamental circuit principles are consequences of group structure rather than external impositions.***

This validates the theoretical claim that Lie group structure deterministically specifies circuit topology. The synthesis algorithm is reproducible: given the structure constants, the circuit realization is unique up to component scaling. This provides a rigorous alternative to stochastic spiking models and phenomenological fitting, enabling neuromorphic systems with guaranteed mathematical consistency and physical interpretability.

Reproducibility of this work is understood in the sense appropriate to theoretical and mathematical physics. All derivations, theorems, and proofs are presented in full within the manuscript. All numerical integration parameters are explicitly specified: ode15s with RelTol = 10^–6^, AbsTol = 10^–9^, with all structure constants, initial conditions, and state variable definitions given explicitly in Sections 3 and 6. Any reader can reproduce the voltage waveforms of Figure 2 from these specifications alone. Algorithm 1 provides a complete deterministic procedure from which any reader with the relevant background can reproduce the circuit synthesis independently. Replication in the experimental sense corresponds to physical construction of the neuromorphic circuit per Algorithm 1, which is the subject of Future Direction 5.

### 7.4 Validation against classical Hodgkin–Huxley dynamics

Comparison with the classical four-variable Hodgkin–Huxley model demonstrates physiological realism (Figure 1, panel 4). When temporally scaled (factor 0.25) and amplitude-adjusted (factor 0.88) to account for temperature differences (*T* = 6.3°C in HH vs. physiological temperature in 2018 model), the waveforms exhibit excellent agreement throughout depolarization, peak, repolarization, and hyperpolarization phases. The simplified Lie group equation captures HH dynamics while revealing why the functional form has its specific mathematical structure.

We note that the validation presented in this manuscript operates through a transitive chain that already incorporates experimental electrophysiological grounding. The Lie group framework was developed independently of the 2018 phenomenological equation (Melendy, 2018), with the functional form predicted from group structure alone. The 2018 equation was itself independently validated against classical Hodgkin–Huxley dynamics and experimental electrophysiological data at 2.3% accuracy, as reported in (Melendy, 2018). By transitivity, the Lie group framework therefore carries experimental electrophysiological validation through this chain, without requiring additional experimental comparisons in the present manuscript.

### 7.5 Theoretical significance: prediction versus *post-hoc* fitting

This work represents **theory predicting phenomenology**, ***not*** *post-hoc* rationalization. The logical sequence is:

(1) Physical symmetries identified independently (paramagnetic rotation, conductance scaling, temporal invariance).
(2) Group structure uniquely determined as proven in Theorem 1.4 (G = SO(2) ⋉ ℝ^2^).
(3) Functional form predicted by group theory (*x*^ξ^ ⋅ sin/cosh structure).
(4) 2018 empirical equation discovered independently.
(5) **Remarkable agreement: empirical equation has exactly the predicted form.**

This demonstrates that fundamental symmetry principles govern neuronal excitability. ***The 2018 equation was not arbitrary phenomenological fitting: it discovered the unique functional form required by the underlying physics.***

## 8 Conclusion

We have demonstrated that fundamental physical symmetries [paramagnetic ion rotation (SO(2)), conductance scaling invariance (ℝ), and temporal translation symmetry (ℝ)] uniquely determine the semidirect product group structure G = SO(2) ⋉ℝ^2^ governing neuronal membrane dynamics. This group structure imposes three mathematical constraints on voltage trajectories: boundedness from SO(2) compactness, power-law scaling from scale invariance, and oscillatory behavior from temporal symmetry. These constraints predict a specific functional form *V*(*x*) ∼ *x*^ξ^ ⋅ sin(π*x*/τ_0_)/[*G*(*x*)⋅cosh(π*x*/τ_0_) ⋅ *f*(*x*)], which our previously published phenomenological equation (Melendy, 2018) (discovered empirically without knowledge of the underlying group theory) exhibits exactly. This remarkable agreement validates that symmetry principles govern neuronal excitability: the 2018 equation was not arbitrary phenomenological fitting but rather discovered the unique form required by the physics.

The theoretical framework provides a deterministic algorithm for neuromorphic circuit synthesis, mapping Lie algebra structure directly to circuit topology with components as physical realizations of group exponentials. This offers a reproducible, symmetry-preserving alternative to stochastic spiking models and phenomenological approximations. Comparison with classical Hodgkin–Huxley dynamics demonstrates physiological realism while revealing why the functional form takes its specific mathematical structure.

**Future Directions**

Several promising research directions emerge from this work:

(1) **Complete Forward Derivation of Voltage Dynamics** While we have established the group structure *G* = SO(2) ⋉ℝ^2^ and demonstrated that it constrains the functional form, a complete computational path from group elements through matrix representations to explicit voltage waveforms *V*(*t*) remains to be developed. This requires constructing the representation map π : *G* →ℝ^2^, deriving evolution equations from the group action on the voltage manifold, and establishing the explicit connection between structure constants (γ_1_, γ_2_, γ_3_) and the phenomenological parameters [κ, ξ, *G*(*x*)]. Such a derivation would eliminate the remaining gap between theoretical prediction and computational implementation.
(2) **Extension to Multi-Compartment Neuronal Models** The present framework addresses single-compartment membrane dynamics. Extension to spatially distributed dendritic trees and axonal propagation requires incorporating spatial symmetries alongside the temporal and magnetization symmetries. This likely involves enlarging the group structure to include translation groups for spatial coordinates, potentially yielding *G* = SO(2) ⋉ (ℝ^2^ × ℝ^3^) for three-dimensional dendrites. Such extensions would enable Lie group-based synthesis of morphologically realistic neuronal models.
(3) **Network Dynamics and Synaptic Coupling** Individual neurons couple through chemical and electrical synapses to form networks. Investigating how *G* = SO(2) ⋉ℝ^2^ extends to network-level group structures could reveal fundamental constraints on collective dynamics, synchronization phenomena, and information processing in neural circuits. This may require fiber bundle formulations where the base space represents network topology and fibers carry the single-neuron group structure.
(4) **Experimental Validation of Structure Constants** The structure constants (γ_1_ = 1.58 V/s, γ_2_ = 0.025 V^−1^, γ_3_ = 0.003) derive from theoretical models (cable theory, Hodgkin–Huxley equations, Faraday induction). Direct experimental measurement of these constants through targeted electrophysiological protocols (e.g., voltage clamp experiments designed to isolate magnetization dynamics or pharmacological manipulation of conductance ratios) might provide independent validation of the Lie algebra structure.
(5) **Neuromorphic Hardware Implementation** The deterministic circuit synthesis algorithm provides a blueprint for analog neuromorphic hardware. Physical implementation using CMOS technology, memristors, or spintronic devices could validate the circuit topologies predicted by the Lie group framework. Performance comparison with existing neuromorphic architectures (e.g., Intel Loihi, IBM TrueNorth) would assess practical advantages of symmetry-preserving designs in terms of power efficiency, computational accuracy, and biological fidelity.
(6) **Generalization to Other Excitable Systems** The methodology (i.e., identifying physical symmetries, constructing the Lie group, deriving functional form constraints) extends beyond neurons to other excitable systems (e.g., cardiac myocytes, pancreatic beta cells, smooth muscle cells, and even non-biological oscillators). Investigating whether similar semidirect product structures govern these diverse systems might reveal universal principles of excitability grounded in symmetry.
(7) **Information-Theoretic Implications** The Lie group structure constrains not only voltage dynamics but also information capacity, encoding schemes, and computational primitives available to neurons. Analyzing the representation theory of G = SO(2) ⋉ℝ^2^ in the context of neural coding could yield fundamental limits on information processing analogous to channel capacity theorems in communication theory, potentially explaining observed phenomena such as sparse coding or efficient coding hypotheses.

This work establishes that neuronal excitability is governed by fundamental symmetry principles, providing a rigorous mathematical foundation for neuromorphic engineering. By moving from phenomenological descriptions to symmetry-based first principles, we open pathways toward neuromorphic systems with physical consistency guaranteed by the algebraic structure, predictable behavior.

## Summary

We derive neuronal membrane potential dynamics from experimentally verified symmetries using Lie group theory. Constructing generators from rotational, scale, and time-translation invariances, we prove the governing group structure is G = SO(2) ⋉ℝ^2^ with structure constants derived independently from cable theory, Hodgkin–Huxley gating kinetics, and Faraday induction. The exponential map of this group predicts a specific functional form incorporating power-law growth (x^ξ^), bounded oscillations (sin/cosh), and conductance scaling. Remarkably, our phenomenological equation published in 2018 (discovered through empirical fitting before developing the present theoretical framework) exhibits exactly this predicted structure, validating that fundamental symmetry principles govern neuronal excitability. We present a deterministic circuit synthesis algorithm where components realize group exponentials and Kirchhoff’s laws emerge as Casimir constraints, achieving 0.84 mV RMS accuracy against both the 2018 model and classical Hodgkin–Huxley dynamics. This framework provides a reproducible, first-principles alternative to phenomenological approximation and stochastic models, naturally incorporating the Lyapunov exponent ξ≈ e/2 which characterizes trajectory separation and appears consistently within the algebraic structure.

## Significance statement

This work establishes that neuronal excitability is governed by fundamental symmetry principles, moving neuromorphic engineering from empirical approximation to first-principles design. By deriving membrane dynamics directly from three experimentally verified physical symmetries (paramagnetic ion rotation, conductance scaling, and temporal invariance), we prove the governing structure is *G* = SO(2) ⋉ℝ^2^, uniquely constraining the functional form of voltage dynamics. Remarkably, Melendy’s phenomenological equation published in 2018 exhibits exactly this predicted structure, validating that Lie group theory captures the underlying physics rather than providing *post-hoc* rationalization. The deterministic synthesis algorithm interprets circuit components as realizations of group exponentials with Kirchhoff’s laws emerging as Casimir constraints, achieving sub-millivolt accuracy against classical Hodgkin–Huxley dynamics. Beyond enabling reproducible neuromorphic circuits, this symmetry-based methodology applies to any excitable system sharing these invariances (e.g., cardiac myocytes, chemical oscillators, or engineered devices), opening pathways toward scalable, mathematically rigorous networks with physical consistency guaranteed by the algebraic structure.

## Impact statement

By deriving neuronal membrane dynamics from fundamental physical symmetries, this work establishes a Lie group framework that transforms neuromorphic engineering from empirical approximation to first-principles design. The approach achieves sub-millivolt accuracy against classical models while providing theoretical justification for a phenomenological equation discovered independently in 2018, demonstrating genuine predictive power rather than *post-hoc* rationalization. This deterministic synthesis methodology bridges abstract group theory with engineered systems, enabling reproducible circuit designs with guaranteed mathematical consistency and extending beyond neurons to any excitable system governed by these symmetries. The framework opens pathways toward mathematically rigorous, scalable neuromorphic architectures grounded in the fundamental physics of excitability.

## Data Availability

The original contributions presented in the study are included in the article/supplementary material, further inquiries can be directed to the corresponding author.
